# Sequence of Age-Associated Changes to the Mouse Neuromuscular Junction and the Protective Effects of Voluntary Exercise

**DOI:** 10.1371/journal.pone.0067970

**Published:** 2013-07-03

**Authors:** Anson Cheng, Marco Morsch, Yui Murata, Nazanin Ghazanfari, Stephen W. Reddel, William D. Phillips

**Affiliations:** 1 Physiology and Bosch Institute, University of Sydney, Sydney, New South Wales, Australia; 2 Department of Molecular Medicine, Concord Hospital, Concord, New South Wales, Australia; Georgia Regents University, United States of America

## Abstract

Loss of connections between motor neurons and skeletal muscle fibers contribute to motor impairment in old age, but the sequence of age-associated changes that precede loss of the neuromuscular synapse remains uncertain. Here we determine changes in the size of neuromuscular synapses within the tibialis anterior muscle across the life span of C57BL/6J mice. Immunofluorescence, confocal microscopy and morphometry were used to measure the area occupied by nerve terminal synaptophysin staining and postsynaptic acetylcholine receptors at motor endplates of 2, 14, 19, 22, 25 and 28month old mice. The key findings were: 1) At middle age (14-months) endplate acetylcholine receptors occupied 238±11 µm^2^ and nerve terminal synaptophysin 168±14 µm^2^ (mean ± SEM). 2) Between 14-months and 19-months (onset of old age) the area occupied by postsynaptic acetylcholine receptors declined 30%. At many endplates the large acetylcholine receptor plaque became fragmented into multiple smaller acetylcholine receptor clusters. 3) Between 19- and 25-months, the fraction of endplate acetylcholine receptors covered by synaptophysin fell 21%. By 28-months, half of the endplates imaged retained ≤50 µm^2^ area of synaptophysin staining. 4) Within aged muscles, the degree to which an endplate remained covered by synaptophysin did not depend upon the total area of acetylcholine receptors, nor upon the number of discrete receptor clusters. 5) Voluntary wheel-running exercise, beginning late in middle-age, prevented much of the age-associated loss of nerve terminal synaptophysin. In summary, a decline in the area of endplate acetylcholine receptor clusters at the onset of old age was followed by loss of nerve terminal synaptophysin from the endplate. Voluntary running exercise, begun late in middle age, substantially inhibited the loss of nerve terminal from aging motor endplates.

## Introduction

There is increasing evidence to suggest that impaired motor innervation contributes to the weakness and impaired mobility of old age. Previous studies in humans have shown that from the age of 60 there were reductions in the number of motor units, with a compensatory expansion of the residual motor units [1], [2], [3]. Studies in rodents suggest loss of motor innervation may contribute to reduced strength by reducing the average force produced per motor unit [4] and by leaving some muscle fibers functionally denervated and atrophied [5], [6]. What changes might be occurring at the aging neuromuscular junction (NMJ) prior to the loss of the neuromuscular connection?

The NMJ is a relay synapse. It is thought to depend upon its large size for proper function. Effective neurotransmission involves simultaneous release of quanta of acetylcholine (ACh) from many sites of exocytosis (spread across the several nerve terminal branches) onto postsynaptic membrane folds that contain tightly clustered acetylcholine receptors (AChR) [7], [8], [9]. Previous reports have described several structural changes to the typical large, pretzel-shaped structure of the NMJ between adulthood and old age. Electron microscopy of post-mortem muscle samples from elderly humans revealed motor endplate postsynaptic gutters devoid of a nerve terminal, while in younger individuals most gutters contained a nerve terminal [10]. Similarly, muscles of aged/geriatric mice contained a greater percentage of endplates that were scored as either 'denervated' or 'partially innervated' compared to younger animals [11], [12], [13].

A second feature of NMJs in aged rodents is the fragmentation/remodeling of postsynaptic specializations. In aged animals the endplate often consisted of many small AChR clusters [11], [14], [15]. A recent study by Li and colleagues repeatedly imaged NMJs on the surface of the mouse sternomastoid muscle as the mouse grew old [16]. This longitudinal analysis showed that the gross structure of any given NMJ remained stable for many months but could then change suddenly, with remodeling/fragmentation of the postsynaptic AChRs into multiple smaller AChR clusters. Interestingly, these sudden endplate-remodeling events coincided with sporadic degeneration and regeneration of the underlying muscle fiber. With age there was an increase in the frequency of these rare muscle fiber degeneration/regeneration events, thereby explaining the accumulation of fragmented endplates in old mice [16].

Muscle usage can influence both pre- and postsynaptic elements of the NMJ over time. In young adult rats, 10 weeks of treadmill training produced an increase the size of the nerve terminal at the motor endplate [17]. The same study showed that 10 weeks of an intervention to reduce muscle usage (suspending the hindlimbs) resulted in a reduction in postsynaptic AChR area, associated with muscle fiber atrophy. Deschenes and Wilson [18] studied the effect of 4 weeks of reduced muscle usage (hindlimb suspension) in aged (22-month old) rats. In the soleus muscle of aged rats hindlimb suspension led to increases in the areas of nerve terminal synapsin and postsynaptic AChR. Valdez and colleagues [11] studied the effect of 4-weeks of voluntary running exercise in aged (22-month) mice. One month of wheel running reduced the percentage of endplates within the gastrocnemius muscle that were classified as either denervated or fragmented. These studies prompted us to further investigate the effect of voluntary running on endplate synaptic area in aging mice.

A number of studies have investigated the effect of age on NMJ structure [11], [12], [18], [19], [20], [21]. The findings have differed somewhat, perhaps reflecting the particular muscle studied and ages compared. Most studies have compared NMJs at just two ages, arbitrarily classified as “old” versus “young” or “adult”. Moreover few of the studies reported the size of the pre- and postsynaptic specializations, thus making quantitative comparisons difficult. This leaves uncertain the sequence of pre- and post-synaptic changes that lead up to the loss of neuromuscular connections. Here we have compared NMJs in the tibialis anterior (TA) muscle at six ages across the lifespan of female C57BL/6J mice. We report a fall in the postsynaptic AChR area at the onset old age, followed by a marked loss of nerve terminal synaptophysin at many endplates. Voluntary running exercise, begun in late middle-age, was sufficient to preserve much of the endplate nerve terminal area that would otherwise be lost.

## Methods

### Ethics Statement

All mouse experiments described in this paper were conducted with the approval of The University of Sydney Animal Ethics Committee (approval number K22/10-2011/3/5602) in compliance with the NSW Animal Research Act 1985 and the Australian Code of Practice for the Care and Use of Animals for Scientific Purposes 7th Edition NHMRC 2004.

**Figure 1:**
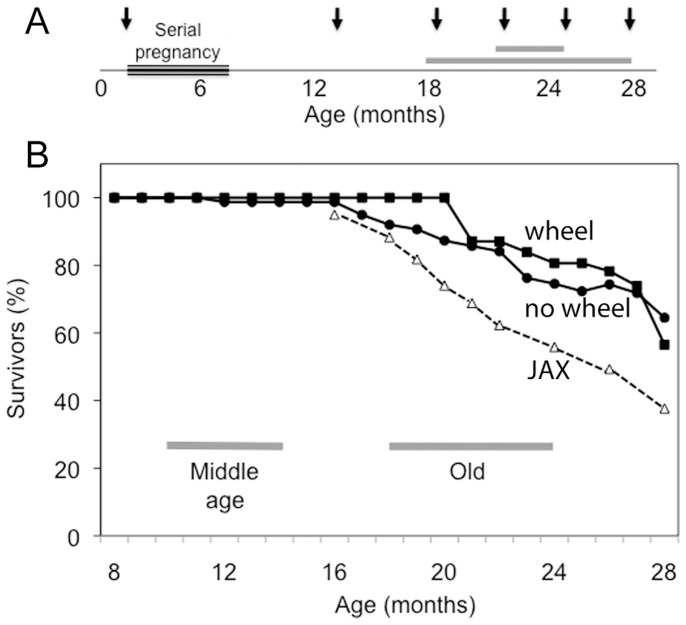
Life history and survival curves for the female C57BL/6J mice used in this study. (**A**) Between 2- and 8-months of age all the mice were engaged in stock breeding. From 8-months mice were group-housed under our care (see *Methods*). Arrows indicate the ages at which mice were killed for analysis. Some boxes of mice received a running wheel from late middle-age until sacrificed (horizontal grey bars). (**B**) Survival curves. Filled circles reflect the percentage of mice in our care that died of natural causes or were euthanized due to disease (starting number 79). Filled squares show results for mice provided access to a running wheel from late middle age (starting number 31). The horizontal grey lines in this panel indicate the age ranges recommended for life-stage comparisons while the dashed line shows comparative survival data for female C57BL/6J mice, both from Jackson Laboratories [25].

Female C57BL/6J mice were obtained from the Animal Resources Centre (Perth, Western Australia) at 2-month of age, or as ex-breeders at 8-months (used for all subsequent time points). All mice were group housed in filter-top cages with wire-grid lids (17×34×13 cm W×L×H; 2–4 mice per box) and were fed rat and mouse chow (YSF brand) *ad libitum*. As a precaution against spread of pathogens sentinel mice, kept on the same rack, were sero-tested for pathogens quarterly by the animal facility veterinarian. The aging mice were inspected twice weekly by the authors and were euthanized if they developed an obvious tumor or sores that did not resolve. Over the course of the study 25% of the aging mice were found dead of natural causes. A further 9% required euthanasia (Fig. 1B). When mice reached the required age (Fig. 1A arrows) they were killed by an overdose of pentobarbitone (30 mg intraperitoneally; CenVet Australia). At the time of dissection an additional 9% of mice were excluded from study due to evidence of disease, according to predetermined criteria based upon published gerontological guidelines [22]. Specifically a mouse was excluded if it displayed abnormalities such as a tumor or a three-fold enlarged spleen (spleen/body weight ratio >0.01).

To test the effect of exercise a low profile wireless running wheel (Med Associates Inc) was placed in the mouse cage. The number of wheel rotations was used to track average distance run over time, dividing the total distance run by the number of mice in the box. Overnight infrared video recordings showed that mice without access to a wheel engaged in some climbing on the underside of the wire cage grille. However sampling of the videos suggested that when aging mice were provided with a running wheel they spent about five-fold more of their time engaged in all exercise-type activities (mainly running), compared to aged mice without a wheel.

### Immunostaining and Confocal Imaging

At the time of sacrifice muscles were immediately dissected, embedded in OCT compound (ProSciTech Australia), snap frozen in liquid nitrogen and stored at −80°C. Cryosections were stained for immunofluorescence as described previously [23], [24]. Briefly, the TA muscle was sectioned longitudinally to the long axis of the fibers (20 µm). Cryosections were collected onto Polysine covered microscope slides (Thermo Scientific, Braunschweig, Germany). After drying the sections were fixed for 15 min in 2% paraformaldehyde/phosphate buffered saline (PBS) at room temperature. They were then washed in PBS containing 0.1 M glycine for 30 min, permeabilized in methanol (−20°C) for 7 min and blocked for 1 hr in PBS containing 0.2% Triton X100 and 2% bovine serum albumin. Slides were washed three times with PBS between every step. Sections were immunolabeled overnight at 4°C with rabbit anti-synaptophysin (1∶200; Dako Australia) or, in some experiments, with a cocktail of anti-synaptophysin and rabbit anti-neurofilament 200 (1∶8000; Sigma). Washing was followed by 1 hr incubation with fluoresceine isothiocyanate (FITC)-conjugated donkey anti-rabbit IgG (1∶250; Jackson Labs). AChRs were stained with Alexa555-BGT-α-bungarotoxin (1∶200, Molecular Probes). Control sections (either no primary or no secondary antibody) were included in every staining experiment to test for non-specific staining and autofluorescence.

Confocal Z-stack images of NMJs were collected by scanning laser microscopy on the Leica SPE II confocal microscope (Bosch Institute, University of Sydney). Endplates were imaged using an oil immersion 63X objective and a zoom factor of 2. The image size was 1024×1024 pixels and scanning was conducted at 600 Hz with the pinhole set to 1 Airy Unit. Z-stacks were collected with a spacing of 0.7 µm. To ensure retention of tonal detail, gain and offset were optimized for synaptophysin and AChR fluorescence at each individual endplate imaged? To reduce photomultiplier noise four scans were averaged. Precautions were taken to reduce the risk of subjective bias in sampling and analysis of NMJs. Within a given immunostaining experiment sections were collected from muscles of young versus old mice, or wheel versus non-wheel mice. Microscope slides were then coded by M.M. Image sampling and subsequent morphometry was then undertaken by A.C. blind to the age/treatment group of each slide. Each section was systematically scanned on the AChR fluorescence channel. All endplates that were relatively flat, presenting en face to the objective (AChR staining extending ≤15 µm in the Z dimension) were individually imaged and morphometrically analyzed.

### Image Analysis

Two dimensional maximum intensity projection images were constructed from Z-stacks using the Leica software. Using NIH ImageJ software (rsbweb.nih.gov/ij/) a line was drawn to enclose all the AChR clusters comprising a single endplate. The area within this perimeter was defined as the total perimeter area. A minimum intensity threshold was then applied to the AChR staining such that the binary (suprathreshold) image included all AChR-rich structures in the endplate as perceived by eye from the full-tone image. This often yielded several discrete suprathreshold AChR clusters per endplate. The area of these AChR clusters was determined using the ‘*analyze particles’* command of ImageJ. Tiny suprathreshold areas consisting of fewer than 15 contiguous pixels (potentially photomultiplier noise) were excluded from the analysis. All remaining suprathreshold areas were summed to yield the total AChR area for the endplate. The minimum threshold method was also used to measure the area of synaptophysin-positive nerve terminal staining at the endplate. The total area of synaptophysin staining was assessed by summing all of the suprathreshold pixels that fell within the total (AChR) perimeter area using the ‘*measure’* tool of ImageJ. At some endplates nerve terminal branches extended slightly beyond the AChR perimeter boundary. Such extensions were included in the measurement of nerve area. The ‘*colocalization’* plug-in of ImageJ was used to create an overlay of the binary AChR and synaptophysin images, yielding the area of overlap.

### Statistics

Comparisons among the means for animals at multiple ages were tested by one-way ANOVA followed by Bonferroni's multiple comparisons post-test. Pair-wise comparison between the means for aged animals with and without a running wheel was by a two-tailed, unpaired Student's t-test. Some of the pooled data comparing endplates at multiple ages did not show a normal distribution so the non-parametric Kruskal-Wallis one-way ANOVA was used followed by Dunn's post-test. For all statistical tests significance was taken as P<0.05.

## Results

Figure 1A represents the lifespan of the female C57BL/6J mice and the ages at which mice were killed for analysis (arrows). With the exception of the two-month old virgin mice, all our mice were ex-breeders. Middle age in C57BL/6J mice has previously been defined as 10- to14-months of age, while old-age is considered to begin at about 18-months [25]. By 28 months of age, 34% of the mice under our care had died of natural causes or had required euthanasia (Fig. 1B filled circles). Survival rates were similar for mice provided with a running wheel from late middle age (Fig. 1B filled squares). This is broadly consistent with published survival curves from Jackson Laboratories, which show about half the female C57BL/6J population surviving until 28-months of age [25], [26]; Fig. 1B JAX, dashed line).

### Age-associated Changes in Nerve Terminal and Postsynaptic AChRs

**Figure 2:**
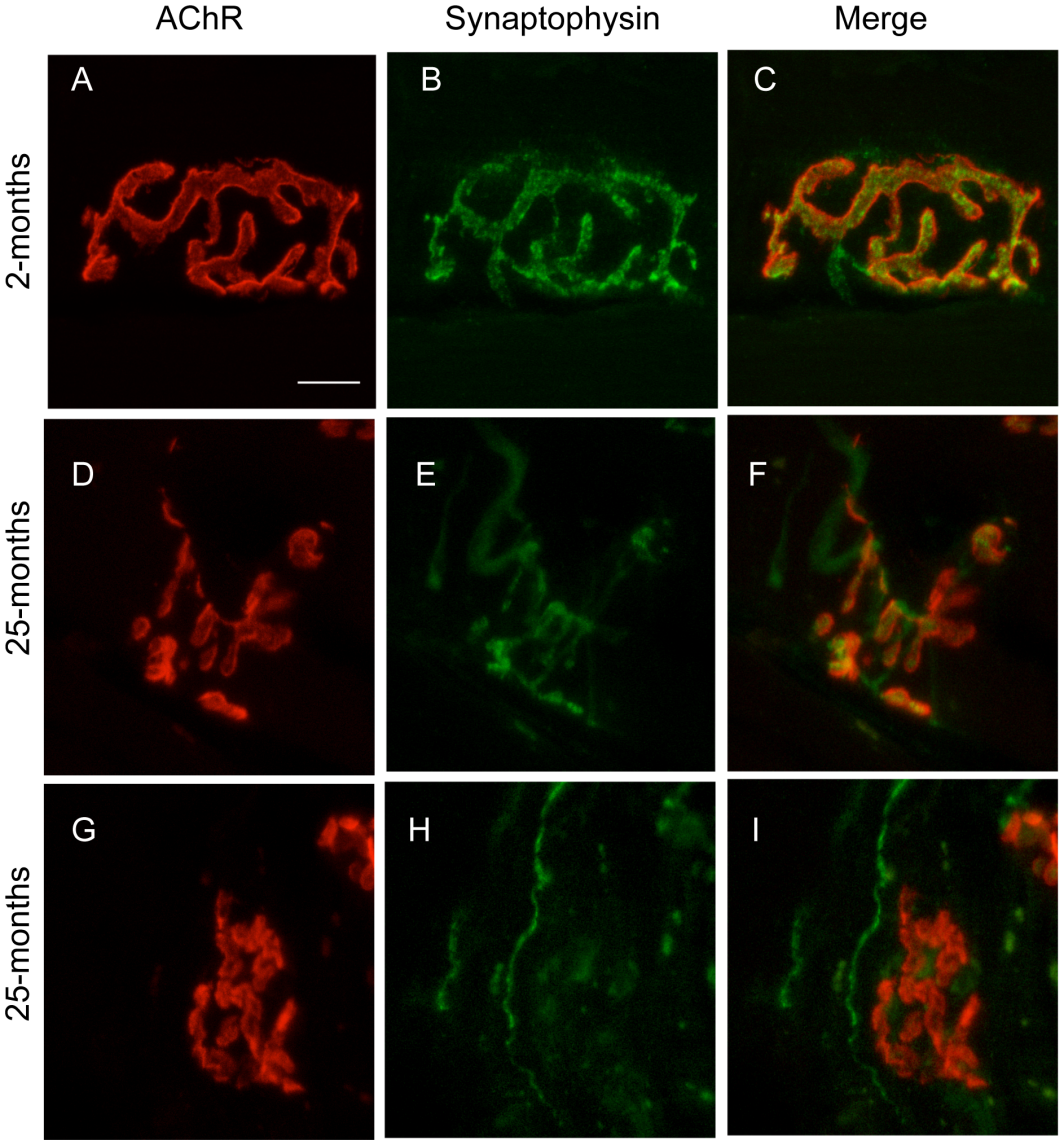
Typical NMJs from the TA muscle of young and old mice viewed en face. Confocal maximum projection images of individual NMJs from the TA muscle were stained for postsynaptic AChR and nerve terminal synaptophysin. (**A–C**) NMJ from a 2-month old mouse showing AChRs (A), synaptophysin (B) and a merge of the pre- and post-synaptic staining (C). (**D–F**) NMJ from a 25-month old mouse at which the AChR-stained primary synaptic gutters were more fragmented but remained largely covered by synaptophysin staining. (**G–I**) NMJ from a 25-month month old mouse at which the AChR-rich area of the endplate was only partially covered by synaptophysin staining. Scale bar in panel A is 10 µm.

Figure 2A is an example of endplate AChR staining from a 2-month old mouse, viewed en face. The densely packed cluster of AChR (red) forms a typical pretzel-shaped plaque composed of branched primary synaptic gutters. The synaptic vesicle protein, synaptophysin, showed a matching pattern of immunostaining (Fig. 2B, green). The merged fluorescence image highlights the close alignment of nerve terminal branches with the AChR-rich primary synaptic gutters (Fig. 2C). Endplates from old mice (25-months) often displayed AChR clusters that were more fragmented and complex in shape (Fig. 2D). Many aged NMJs revealed portions of the endplate AChR clusters that had little or no overlying synaptophysin staining (Fig. 2G–I).

**Figure 3:**
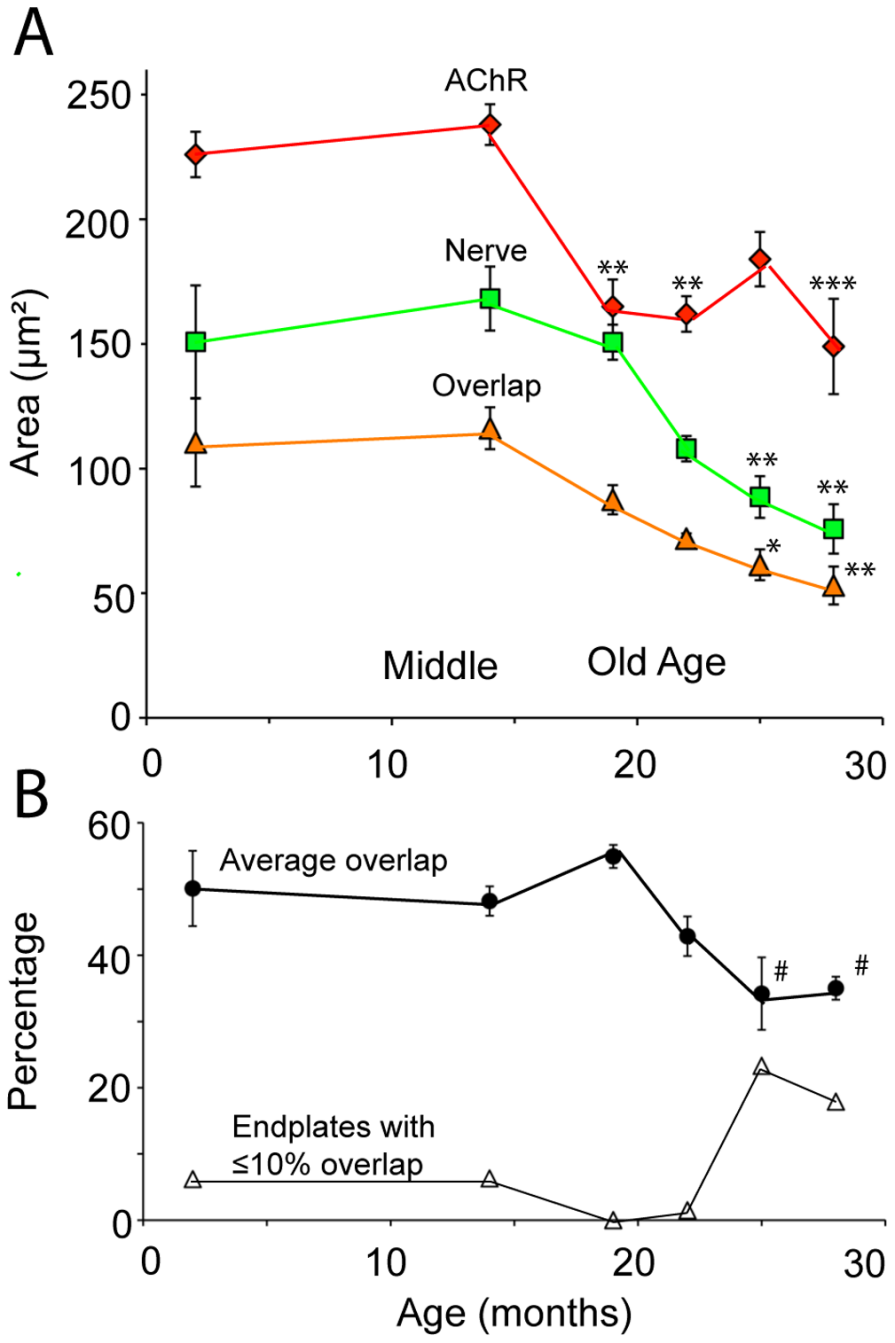
Changes with age in the area of nerve terminal synaptophysin and postsynaptic AChRs. (**A**) Average area of nerve terminal synaptophysin staining (Nerve; green squares) and postsynaptic AChRs (red diamonds) with age. The area of overlap between synaptophysin and AChR staining (synaptic overlap) is indicated by amber triangles (* P<0.05, ** P<0.01, *** P<0.001 relative to 14-month mice). (**B**) Changes in the area of synaptic overlap, expressed as a percentage of the endplate AChRs area (filled circles; # P<0.05 relative to 19-month old mice). Filled symbols in A and B show the mean ± SEM for n = 3mice (19-months) or n = 4 mice (2-, 14-, 22-, 25- and 28-months). The full list of significant differences by one-way ANOVA with Bonferroni's multiple comparison post-test were as follows. AChR area: 2- vs. 14- and 22-months (P<0.05); 2- vs. 28-months, 14- vs. 19- and 22-months (P<0.1); 14- vs. 28-months (P<0.001). Nerve area: 2- vs. 25-months and 19- vs. 28-months (P<0.05); 2- vs. 28-months, 14- vs. 25- and 28-months (P<0.01). Overlap area: 2- vs. 25-months, 14- vs. 25-months (P<0.05); 2- vs. 28-months, 14- vs. 28-months (P<0.01). Percentage overlap: 19- vs. 25- and 28-months (P<0.05). The decline in average synaptic overlap could be explained, in part, by an increase in the percentage of endplates with ≤ 10% synaptic overlap (from pooled data).

At middle age (14-months) endplate AChR occupied 238±11 µm^2^ while the average area of nerve terminal synaptophysin was 168±14 µm^2^ (mean ± SEM). Between 14- and 19-months (onset of old-age), there was a 30% fall in the average postsynaptic AChR area (Fig. 3A, red diamonds; P<0.01, one-way ANOVA). Beyond 19-months there were no further significant changes in AChR area. Between 14- and 28-months the decline of postsynaptic AChR was followed by a drop in the average area of the synaptophysin-stained nerve terminal (Fig. 3A green squares; P<0.01). The area of overlap between synaptophysin and postsynaptic AChR declined steadily throughout this period (Fig. 3A, orange triangles; P<0.01). Expressed as percentage of AChR area, overlap fell from 55% at 19-months to 34% at 25-months (P<0.05; Fig. 3B filled circles). Thus the decline in synaptic overlap area in old age was associated with sequential reductions in the areas of postsynaptic AChR and presynaptic nerve terminal.

**Figure 4:**
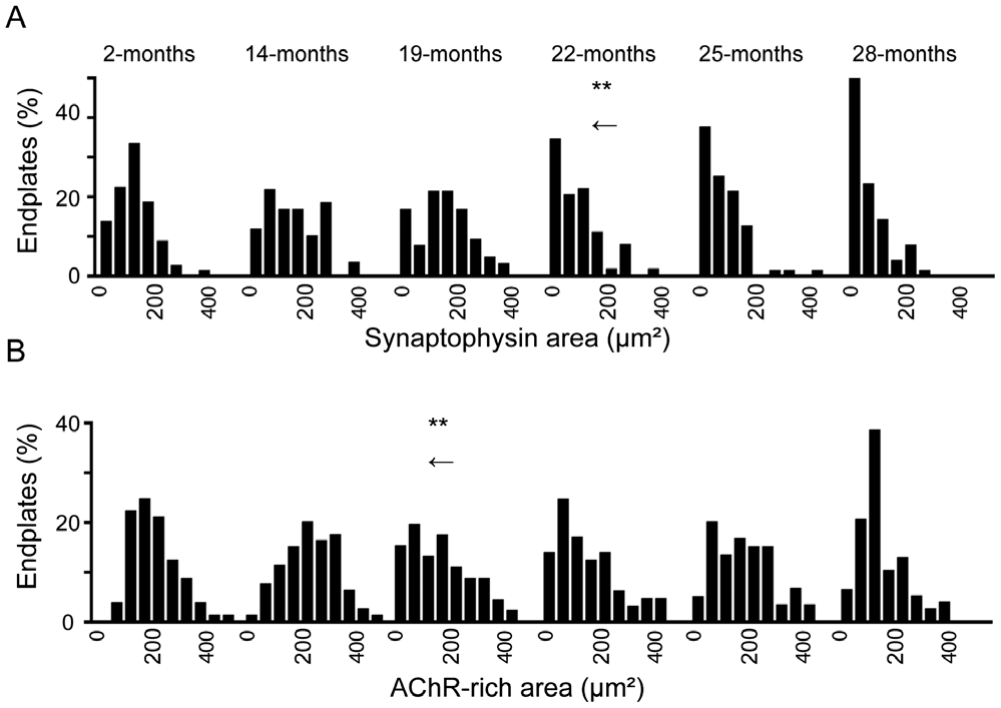
A subset of endplates lose nearly all their synaptophysin in old age. (**A**) Frequency distributions show the synaptophysin-positive nerve terminal area of endplates at 2, 14, 19, 22, 25 and 28 months of age. Note the leftward shift toward endplates with ≤50 µm^2^ synaptophysin area from 22-months onwards. (**B**) Frequency distributions for the AChR-rich area of the endplate. Data represent 60 - 80 NMJs pooled from 3–4 mice at each age. Arrows indicate a statistically-significant shift compared to the immediately-preceding age (**P<0.01; Kruskal-Wallis test followed by Dunn's multiple comparisons post-test). The full set of significant differences were as follows. Synaptophysin area: 2- vs. 25-months (P<0.05); 14- vs 22- and 19- vs. 22- (P<0.01); 2- vs. 28-, 14- vs. 25-, 14- vs. 28-, 19- vs. 25-, 19- vs. 28- (P<0.001). AChR area: 2- vs. 22-months and 14- vs. 19- (P<0.01); 2- vs. 28-, 14- vs. 22-, 14- vs. 28- (P<0.001).

Frequency distributions of pooled endplate data were examined to see whether all NMJs were uniformly affected by age (Fig. 4). At 2-, 14- and 19-months of age the distribution of nerve terminal areas was roughly Gaussian. However from 22-months onwards the distribution of nerve terminal area was strongly skewed to the left, such that many endplates retained little or no synaptophysin-positive nerve terminal (Fig. 4A, note the first bin of each distribution). The Kruskal-Wallis test followed by Dunn's multiple comparisons post-test confirmed a significant leftward shift between the 19- and 22-months distributions (Fig. 4B, asterisks and arrow). By 28-months, half of the endplates sampled retained ≤50 µm^2^ of synaptophysin-positive nerve terminal. At 2- and 14-months the distribution of endplate AChR area was also roughly Gaussian. The 19-month distribution revealed a transient shift to the left (Fig. 4B, asterisks and arrow) reflecting the drop in the mean at this age (Fig. 3A). Thus, with the progression into old age a subset of endplates retained little or no nerve terminal (≤10% of endplate AChR retaining overlapping synaptophysin; Fig. 3B open triangles).

**Figure 5:**
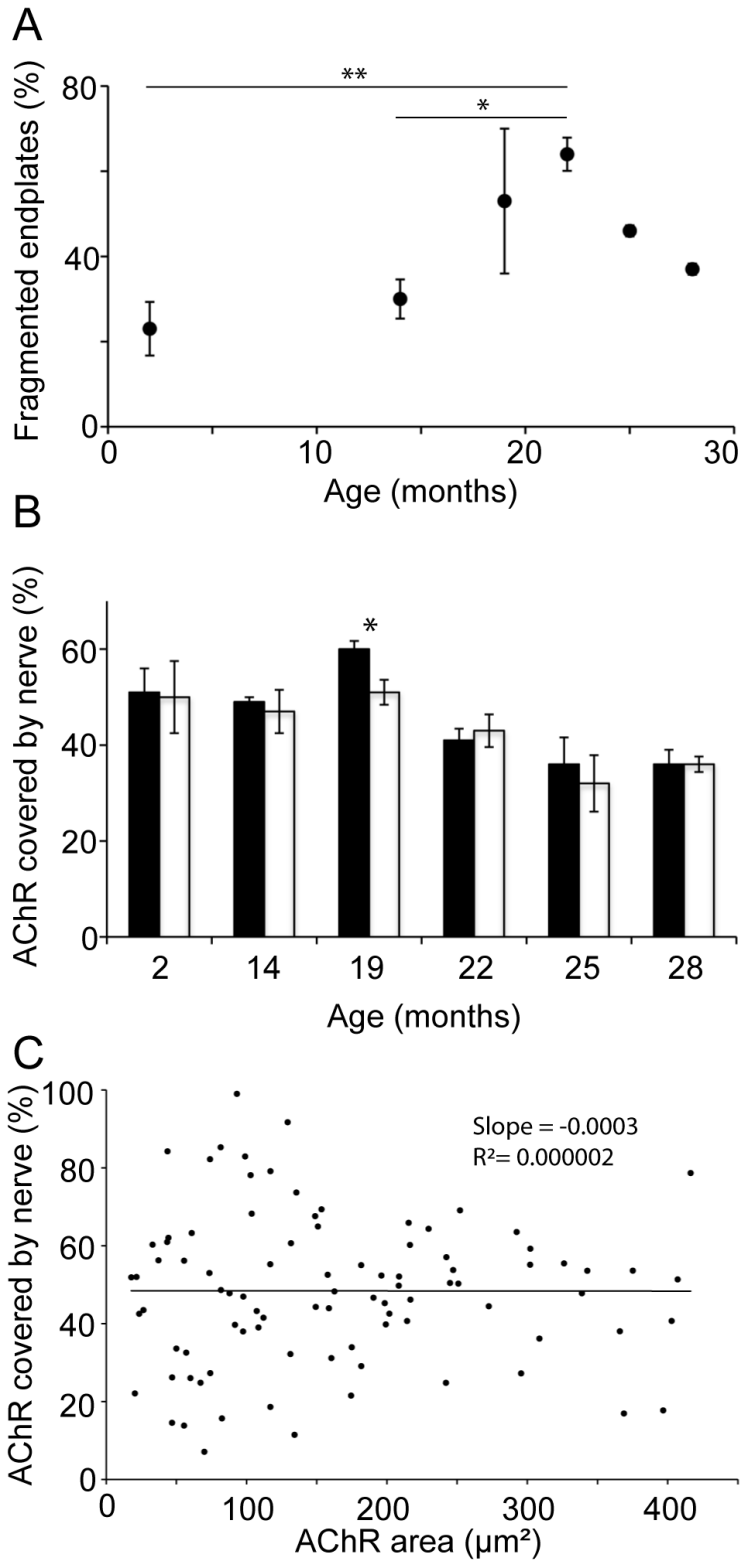
Age-related nerve terminal shrinkage correlates poorly with AChR changes. (**A**) The percentage of endplates composed of five or more discrete AChR clusters (fragmented endplates) increased with the onset of old age (mean ± SEM for n = 4 mice; *P<0.05, **P<0.01 by one-way ANOVA followed by Bonferroni's multiple comparisons post-test). (**B**) The percentage AChR covered by nerve (synaptophysin) was similar for fragmented endplates (open bars) and non-fragmented endplates (1–4 AChR clusters; filled bars). The only significant difference was at 19-months (*P<0.05, unpaired Student t-test). (**C**) Scatter plot comparing the percentage of the AChRs covered by synaptophysin staining to the total area of AChRs at an endplate. Each symbol represents an individual endplate from a 25-month old mouse. No correlation was found between the postsynapic area occupied by AChRs and the extent to which the AChRs remained covered by synaptophysin staining.

With age there was an increase in the proportion of endplates comprised of multiple AChR clusters. Most young endplates consisted of just one or a few large AChR clusters (Fig. 2A) but by 25-months endplates often consisted of many small AChR clusters (Fig. 2D). Fragmented endplates were defined as those composed of five or more AChR clusters. The incidence of these fragmented endplates increased between 14- and 22-months of age (P<0.05; Fig. 5A). We compared the extent to which fragmented and non-fragmented endplates retained nerve terminal staining. For every age except 19-months fragmented endplate AChRs displayed similar coverage with synaptophysin as non-fragmented endplates of the same age (Fig. 5B). In aging mice the total area of AChRs varied widely from endplate to endplate. However we found no correlation between the area of AChRs at an endplate and the percentage covered by synaptophysin (Fig. 5C). Thus there was little to suggest that fragmentation or shrinkage of the postsynaptic AChR clusters predisposes an endplate to loss of nerve terminal synaptophysin.

### Effect of Running Wheel Intervention

**Figure 6:**
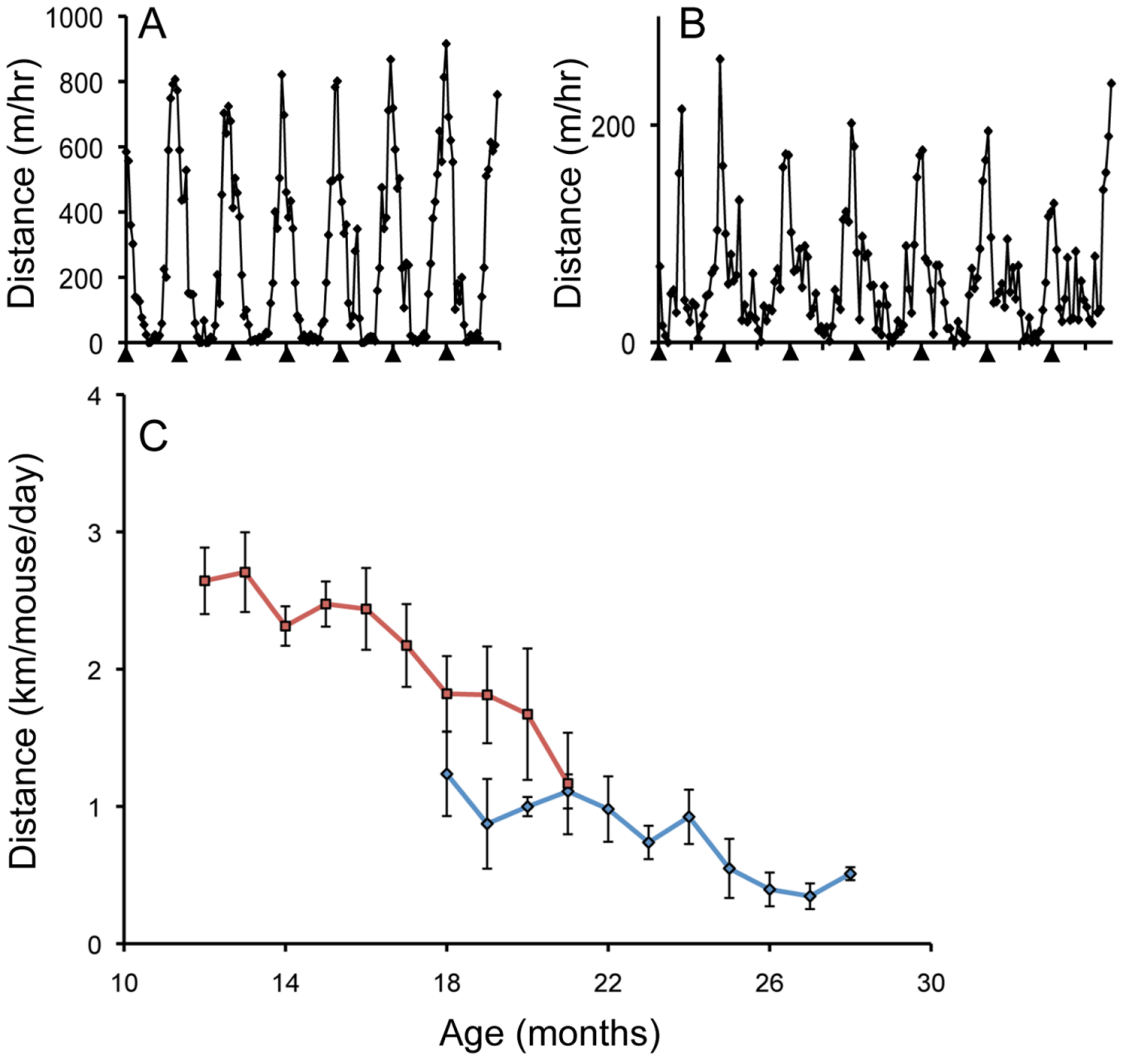
Voluntary wheel running by aging mice. (**A**) One week of typical circadian running activity by 12-month old mice (arrowheads indicate midnight). Distances run are expressed as the average per mouse. (**B**) Circadian running activity by 27-month old mice. (**C**) Decline with age in the average daily distance run per mouse. Blue diamonds show results for mice that received a wheel at 18-months of age. Red squares show results for mice that received a wheel at 10-months of age (the latter mice were not included in the NMJ analysis). Data represents the mean ± SEM for 3–5 boxes of mice at each time-point.

Mice provided with a running wheel engaged in a regular pattern of nocturnal running both in middle- and old-age (Fig. 6A & B respectively). The main difference was that the average daily distance run per mouse declined steadily between 14- and 26-months of age (Fig. 6C), consistent with earlier findings in aging rats [27]. Notwithstanding this decline, 28-month old mice continued to run an average of about 0.5 km/day.

**Figure 7:**
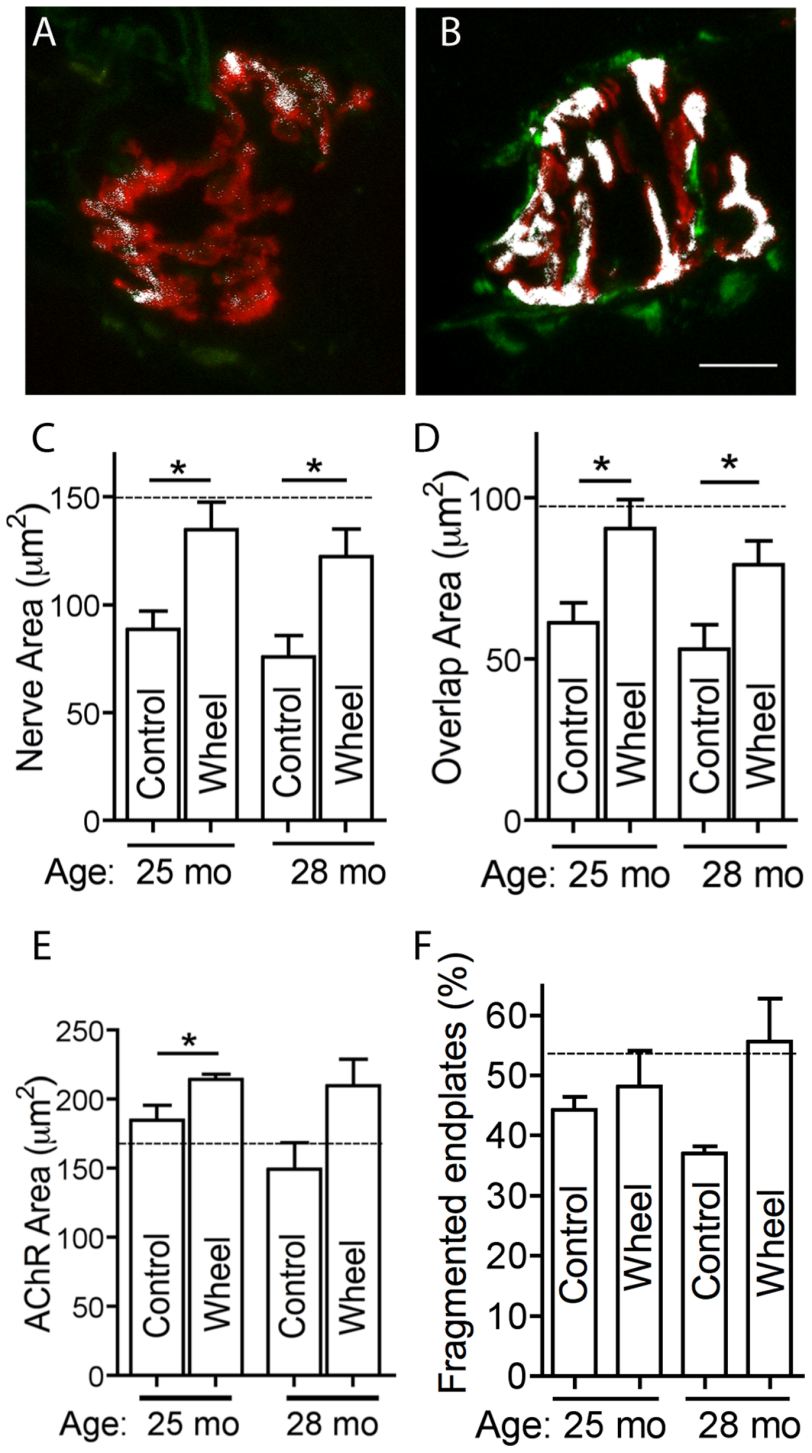
Effect of voluntary wheel running upon the aging NMJ. (**A & B**) Sample endplate from a 25-month old sedentary mouse (A) and from a 25-month old mouse provided with a running wheel from 21-months of age onward (B). Red fluorescence shows AChRs, green fluorescence shows nerve staining and white pixels indicate the area of overlap. Scale bar  = 10 µm. (**C**) The effect of wheel running upon the area of nerve terminal synaptophysin for mice analyzed at 25- and 28months of age (Nerve area). (**D**) The effect of wheel running upon the area of overlap between AChR and synaptophysin staining. (**E**) The effect of wheel running upon the area of endplate AChRs. (**F**) Wheel running did not significantly alter the percentage of endplates composed of five or more AChR clusters (fragmented endplates). Data represent the mean ± SEM (n = 4 control mice, n = 5 wheel mice; P<0.05, unpaired 2-sided Student t-test). The horizontal dashed line in panels C–F shows the mean value for 19-month old mice that did not have access to a wheel (data replotted from Figs. 2 and 5A).

The influence of voluntary exercise on the aging NMJ was evaluated in two experiments. In the first experiment mice received a running wheel at 21-months of age and were killed for analysis at 25-months. In the second experiment mice received a wheel at 18-months and were killed for analysis at 28-months (Fig. 1A). For aged mice that had been provided with a running wheel endplate AChRs were relatively well covered by synaptophysin (Fig. 7B: overlap is shown as white pixels). Indeed for both experiments the nerve terminal area and the area of synaptic overlap were significantly larger for endplates of running wheel mice, compared to age-matched sedentary mice (Fig. 7C and 7D). For 25-month old running wheel mice the mean area of the nerve terminal and the area of synaptic overlap were each comparable to mean values found in 19-month old control mice (dashed horizontal lines in Figs 7C & 7D). For the 25-month-old mice wheel running also produced a modest increase in endplate AChR area, compared to age-matched control (no wheel) mice (Fig. 7E). Wheel running did not significantly affect the percentage of endplates composed of five or more AChR clusters (fragmented endplates; Fig. 7F). Thus exercise prevented much of the loss of nerve terminal area that otherwise occurred in old age.

## Discussion

By measuring pre- and post-synaptic changes to the NMJ in the mouse TA muscle at multiple ages we show here that the transition into old-age is marked by reduction in the size of the neuromuscular synaptic contact. Decline in nerve terminal area was preceded by fragmentation and shrinkage of postsynaptic AChR clusters. Importantly mice provided with a running wheel retained much of their endplate nerve terminal cover well into old age. These results suggest that the loss of neuromuscular synaptic contact is a particular consequence of sedentary aging, and it might be substantially delayed by voluntary exercise.

Loss of nerve endings has been reported at motor endplates in both rodents and humans. In the soleus muscle of aged (22-month old) rats, both pre- and post-synaptic specializations were significantly smaller than for young (8-month old) rats [18]. Chai et al. reported that 20% of the endplates in the extensor digitorum longus muscle of 29-month old mice were denervated [12]. Valdez et al. scored increased numbers of denervated fibers and partially innervated fibres with old age in a number of different mouse muscles [11], [13]. Our approach has been to compare the size of pre- and post-synaptic specializations at NMJs in the TA muscle over various time points throughout the life-span of C57BL/6J mice. In young adult mice synaptophysin-staining covered about half the area of the postsynaptic AChR cluster, consistent with our previous studies [23]. This remained the case even at 19-months of age. The percentage overlap then declined with the onset of old age. For about one fifth of the endplates in aged mice ≤10% of the AChRs remained covered by synaptophysin (Fig. 3B). Most of the latter endplates appeared to retain a tiny area of synaptophysin staining. Whether this residual fluorescence constituted a 'nerve terminal foothold' remains uncertain. The present results demonstrate a marked reduction in the average size of the synaptophysin-positive nerve terminal during the progression into old age.

What is the significance of the fragmentation and shrinkage of postsynaptic AChR clusters? Previous rodent studies found that in old age the large endplate AChR plaque tended to become fragmented into a multiple smaller AChR clusters [11], [14], [15]. Repeated imaging of the same NMJ on the surface of the sternomastoid muscle over several months in living mice has recently provided an important insight [16]. These authors showed that reorganization of AChR plaque into multiple fragments was an occasional, sudden event that followed the degeneration of the underlying muscle fiber. The increased prevalence of fragmented endplates in old age was attributed to an increased incidence of sporadic muscle fiber degeneration events as the animal grew old. While most endplates in the sternomastoid muscle of aged mice were classified as fragmented/remodelled, AChR cluster fragmentation in this muscle did not seem to be associated with loss of nerve terminal staining [13], [16]. This is consistent with the finding that different muscles in the body can vary in the extent to which their endplates lose their nerve terminals in old mice [13]. In the TA muscle we also found an increase in prevalence of fragmented endplates by 22-months of age (Fig. 5A, compare with Fig. 2 of [16]). In aged mice we observed many examples of fragmented endplate AChR clusters that retained abundant synaptophysin coverage (Fig. 2D–F). There was little difference in the extent to which fragmented and non-fragmented endplates retained synaptophysin overlap (Fig. 5B). Some endplates in aged mice showed only a small area of AChR staining, but there was no tendency for endplates with small AChR area to show less synaptophysin overlap than those with a large AChR area (Fig. 5C). In young mice, reinnervation and regeneration of motor endplates following nerve crush leads to an increase in the number of AChR clusters per endplate [28]. The appearance of multiple AChR clusters at endplates in aging mice might likewise reflect adaptive remodeling following reinnervation, without necessarily implying an impaired synaptic relationship. Exercise did not reduce the number of fragmented endplates (Fig. 7F), despite inhibiting the loss of nerve terminal synaptophysin. Taking all of these observations into account it seems unlikely that the processes that lead to the age-related remodeling of the postsynaptic AChR cluster are a cause of the subsequent decline in the size of the nerve terminal.

The most surprising result from the present study was the impact of voluntary running exercise. Early studies in which 28-month old mice were subjected to 3months of treadmill endurance exercise revealed reductions in the size of nerve terminals (stained with zinc iodide-osmium) [29]. Valdez et al. reported that voluntary running wheel exercise reduced the proportion of structurally denervated endplates in the gracilis and medial gastrocnemius muscles. The reductions they found in the TA muscle did not reach significance [11]. Using a longer period of voluntary wheel running (4- or 10-months) and focusing upon the TA muscle we found that most of the age-associated loss of nerve terminal area was prevented. These results raise the intriguing possibility that the age-associated loss of nerve terminals in hindlimb muscles might be substantially delayed by voluntary running.
